# Implante de Válvula Aórtica Transcateter (TAVI) versus Substituição Cirúrgica de Válvula em Resultados Clínicos em Pacientes com Estenose Aórtica

**DOI:** 10.36660/abc.20240295

**Published:** 2025-10-28

**Authors:** André Luiz Lisboa Cordeiro, Emmanuel de Souza Gonçalves, Rute Macêdo de Santana, Tayane Siqueira Martins dos Santos

**Affiliations:** 1 Centro Universitário de Excelência Feira de Santana BA Brasil Centro Universitário de Excelência, Feira de Santana, BA – Brasil; 2 Escola Bahiana de Medicina e Saúde Pública Salvador BA Brasil Escola Bahiana de Medicina e Saúde Pública, Salvador, BA – Brasil

**Keywords:** Estenose da Valva Aórtica, Substituição da Valva Aórtica Transcateter

## Abstract

A estenose aórtica (EA) é uma das lesões valvares mais prevalentes, e a substituição do implante transcateter da valva aórtica (TAVI) surgiu como uma alternativa à substituição cirúrgica da valva aórtica (SAVR). O TAVI é um procedimento minimamente invasivo que se mostrou uma opção mais segura em diversos aspectos. O objetivo é revisar o impacto do TAVI em comparação à SAVR na mortalidade, complicações pós-operatórias, hospitalização e qualidade de vida em pacientes com EA. Foi realizada uma revisão sistemática utilizando a estratégia PICO, com buscas nas bases de dados PubMed, Central e LILACS, empregando os seguintes descritores: EA, hospitalização, mortalidade, ensaio clínico, TAVI, qualidade de vida, complicações pós-operatórias, combinados com os operadores booleanos “AND” e “OR”. Um total de 29 artigos foram encontrados após a leitura dos títulos e resumos. Destes, nove mostraram menor mortalidade em pacientes submetidos ao TAVI, enquanto três relataram menor mortalidade no grupo SAVR. Oito artigos apresentaram achados semelhantes em relação às complicações, com seis mostrando menor incidência de complicações pós-operatórias no TAVI e três no SAVR. Além disso, três artigos mostraram melhor qualidade de vida em pacientes submetidos ao TAVI, enquanto um estudo indicou menor tempo de internação hospitalar para pacientes submetidos ao TAVI. Em todos os estudos, os pacientes analisados tinham mais de 70 anos. O TAVI reduziu a mortalidade em comparação com a SAVR em pacientes com EA. Além disso, o TAVI foi associado à redução da internação hospitalar e à melhora da qualidade de vida. Em relação às complicações pós-operatórias, os resultados indicaram que o TAVI tende a apresentar menor taxa de complicações, embora existam variações entre os estudos.

## Graphical abstract

**Figura Central:**
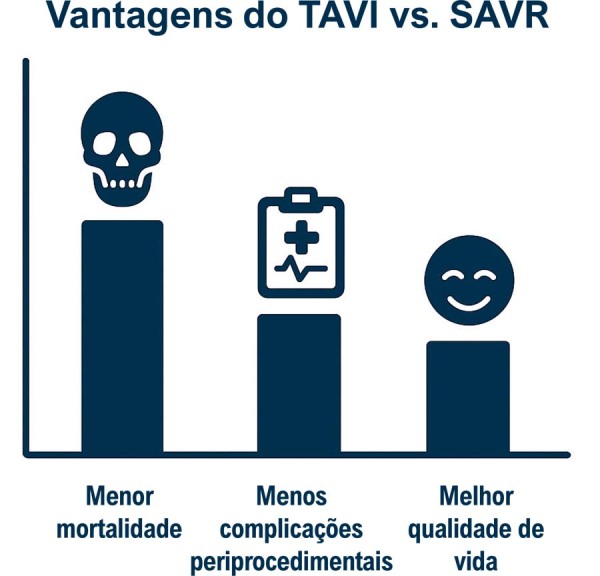
Implante de Válvula Aórtica Transcateter (TAVI) versus Substituição Cirúrgica de Válvula em Resultados Clínicos em Pacientes com Estenose Aórtica

Síntese dos achados obtidos por meio dos artigos analisados.

## Introdução

O procedimento de implante transcateter de válvula aórtica (TAVI) foi realizado pela primeira vez em 2002, representando um grande avanço no tratamento da estenose aórtica (EA). Desde o seu início, a técnica transformou o tratamento da EA, inicialmente desenvolvida para pacientes cirúrgicos de alto risco, mas gradualmente se expandindo para pacientes de risco intermediário e até mesmo baixo, fornecendo uma alternativa minimamente invasiva à substituição cirúrgica convencional da válvula aórtica (SAVR). Estudos demonstraram que o TAVI é eficaz em pacientes de alto e baixo risco cirúrgico, resultando em melhorias significativas nos desfechos clínicos, incluindo redução da mortalidade e morbidade associadas à EA grave.^1 , 2^ O TAVI envolve o implante de um stent, que pode ser autoexpansível, no anel aórtico, coberto por três folhetos internos (tipicamente feitos de pericárdio bovino ou suíno), que funcionam como válvulas semilunares. O acesso para o procedimento é tipicamente via cateterização das artérias femoral ou subclávia.

Nos últimos 20 anos, houve um desenvolvimento contínuo de novas tecnologias e próteses para o procedimento TAVI, resultando em maior confiabilidade no implante valvar e melhores resultados a longo prazo.^3^ A experiência do operador também desempenhou um papel fundamental no sucesso do TAVI e, combinada com os avanços nas técnicas de imagem e no planejamento do procedimento, facilitou a crescente adoção do TAVI para o tratamento da EA. Atualmente, o TAVI representa aproximadamente 12,5% de todas as substituições de valva aórtica realizadas em todo o mundo.^4^ Além disso, estudos demonstraram que, em pacientes selecionados, o TAVI produz resultados superiores em comparação à SAVR, particularmente quando realizado por acesso transfemoral.^3 , 5^

Apesar dos benefícios comprovados do TAVI, persistem incertezas quanto a alguns desfechos importantes do procedimento. Por exemplo, a ocorrência de acidente vascular cerebral (AVC) continua sendo uma preocupação significativa, visto que o posicionamento e a implantação da válvula podem aumentar o risco de eventos embólicos.^6^ Além disso, a comparação da mortalidade entre TAVI e SAVR permanece controversa, com questionamentos em torno da validade de ferramentas como o Índice de Risco da Sociedade de Cirurgiões Torácicos (STS), que ainda não foi totalmente estabelecido como um indicador confiável em todos os casos.^7^ Essas questões ressaltam a necessidade de uma avaliação crítica e completa dos dados clínicos disponíveis para melhor compreender os desfechos associados ao TAVI e sua real aplicabilidade em diferentes populações de pacientes.

Assim, esta revisão sistemática tem como objetivo analisar e comparar os dados disponíveis sobre os desfechos clínicos entre TAVI e SAVR, com o objetivo de esclarecer aspectos que permanecem indefinidos, como mortalidade, complicações pós-operatórias, tempo de hospitalização e qualidade de vida. Com isso, esta revisão busca esclarecer o verdadeiro impacto do TAVI nos desfechos clínicos e fornecer uma base de evidências mais robusta para subsidiar a tomada de decisões na prática clínica.

## Métodos

### Protocolo e registro

Esta revisão sistemática foi concluída de acordo com as diretrizes *Preferred Reporting Items for Systematic Reviews and Meta-analyses* (PRISMA).^8^ Está registrada no PROSPERO (Registro Prospectivo Internacional de Revisões Sistemáticas) sob o número CRD42021218265.

### Critérios de elegibilidade

Esta revisão sistemática seguiu a estratégia PICOS^9^ para estruturar a análise de estudos relevantes. A população do estudo foi composta por pacientes com diagnóstico de disfunção aórtica, especificamente aqueles com estenose ou regurgitação aórtica, candidatos à intervenção. A principal intervenção avaliada foi o TAVI, um procedimento menos invasivo em comparação às opções cirúrgicas tradicionais. O TAVI foi comparado diretamente aos tratamentos convencionais, que tipicamente envolvem esternotomia mediana ou toracotomia para substituição da válvula aórtica, ambas abordagens cirúrgicas mais invasivas.

A revisão se concentrou em vários desfechos importantes: tempo de internação hospitalar, tempo de internação na unidade de terapia intensiva (UTI), qualidade de vida após o procedimento, taxas de mortalidade e ocorrência de complicações pós-operatórias, incluindo, entre outras, AVC, sangramento e problemas relacionados à válvula. Esses desfechos foram selecionados devido à sua relevância clínica na determinação da eficácia e segurança gerais da TAVI em comparação com intervenções cirúrgicas tradicionais.

Apenas ensaios clínicos randomizados (ECRs) foram incluídos para garantir o mais alto nível de evidência para as comparações. Não houve restrições quanto ao ano ou idioma de publicação, permitindo uma inclusão abrangente de estudos em diferentes períodos e regiões. No entanto, para melhorar a precisão da revisão, uma maior elaboração dos critérios específicos de inclusão e exclusão dos estudos fortaleceria a transparência do processo de seleção. Isso ajudaria os leitores a entenderem o escopo das evidências, a generalização dos resultados e quaisquer limitações potenciais relacionadas aos estudos selecionados, como tamanho da amostra ou qualidade metodológica. Além disso, esclarecer como os estudos foram avaliados quanto a viés e risco de falhas metodológicas ofereceria insights valiosos sobre a confiabilidade dos resultados apresentados na revisão.

### Fontes de informação

Realizamos uma busca informatizada, consultando o PubMed, o Registro Central de Revisões Sistemáticas (CENTRAL) e a LILACS. Também pesquisamos a lista de referências de revisões sistemáticas e ensaios clínicos anteriores elegíveis para esta revisão. A busca por artigos terminou em março de 2024.

## Busca

A busca foi baseada na estratégia PICOS^9^ descrita anteriormente e nos operadores booleanos AND e OR. Utilizamos como descritores para a população (Estenose Aórtica ou Estenoses da Valva Aórtica ou Estenoses, Aórtica ou Estenoses, Válvula Aórtica ou Estenose, Aórtica ou Estenose, Válvula Aórtica ou Estenoses de Válvula, Aórtica ou Estenose da Válvula Aórtica) com disfunção aórtica. Para a intervenção (Implante de Válvula Aórtica Transcateter ou Substituição de Válvula Aórtica Transcateter) com TAVI. Para comparação (tratamento convencional ou esternotomia ou toracotomia). Para os resultados (Hospitalizações ou Estadia Hospitalar ou Estadias Hospitalares ou Duração da Estadia ou Duração da Estadia ou Estadia, Hospital ou Estadias, ou Unidades de Terapia Intensiva ou UTI Unidades de Terapia Intensiva ou Unidade, Terapia Intensiva ou Unidades, Terapia Intensiva ou Qualidade de Vida ou Qualidade de Vida Relacionada à Saúde ou Saúde-Qualidade de Vida Relacionada à Saúde ou HRQOL ou Qualidade de Vida ou Taxa de Mortalidade Específica por Idade ou Taxa de Letalidade de Casos ou CFR ou Taxa de Mortalidade Bruta ou Taxa de Mortalidade ou Taxas de Mortalidade, específica por idade ou declínio, mortalidade ou declínio, mortalidade ou determinante, mortalidade ou taxa de mortalidade ou taxa de mortalidade ou mortalidade, Diferencial ou Mortalidade, Excesso ou Taxa, Morte ou Taxa Específica por Idade, Fatalidade ou Taxa de Casos, Morte ou Taxa Bruta, Mortalidade ou Taxa Bruta, Morte ou Taxa, Mortalidade ou Taxas, Morte ou Taxa Específica por Idade, Fatalidade ou Taxas, Morte ou Taxas, Mortalidade ou Mortalidade ou Complicações Pós-Operatórias ou Complicação, Pós-operatório ou Complicações, Pós-operatório) foram por tempo de internação hospitalar e UTI; qualidade de vida; mortalidade e complicações pós-operatórias. Como descritores para o delineamento do estudo (Ensaio Clínico ou Estudo de Intervenção ou Ensaio Clínico Controlado ou Ensaio Controlado Randomizado), utilizamos ECRs, ensaios clínicos e ensaios controlados. A estratégia de busca desenvolvida é apresentada na forma de um suplemento.

### Seleção de Estudos

Esta revisão sistemática incluiu ECRs envolvendo pacientes submetidos a TAVI. Para serem elegíveis, os ensaios clínicos deveriam envolver pacientes com disfunção aórtica submetidos a TAVI. Foram incluídos estudos com adultos (18 anos ou mais), independentemente do sexo. Os critérios de exclusão foram estudos envolvendo pacientes em cuidados paliativos, com expectativa de vida inferior a um ano ou com baixa probabilidade de melhora na qualidade de vida com o tratamento.

Processo de coleta de dados

A extração dos artigos selecionados foi realizada em três etapas sequenciais:

1. **Primeira Etapa** : Verificar os títulos dos estudos para identificar a relevância.
2. **Segunda Etapa** : Analisar os resumos dos artigos para uma pré-seleção mais detalhada.
3. **Terceira Etapa** : Leitura completa dos artigos para avaliação aprofundada.

Após a seleção, foi realizada uma leitura exploratória dos estudos, seguida de uma leitura seletiva e analítica. Os dados extraídos dos artigos foram organizados e sumarizados em termos de: autores, periódico, ano de publicação, título e conclusões. Isso permitiu a coleta das informações mais relevantes para a pesquisa.

### Itens de dados

Três autores (EG, RM e TS) extraíram os dados dos relatórios publicados de forma independente, usando extração de dados padrão, considerando: (1) aspectos da população do estudo, como idade média, sexo, número de pacientes, diagnóstico; (2) aspectos da intervenção realizada; (3) acompanhamento; (4) perda de acompanhamento; (5) medidas de desfecho; e (6) resultados apresentados.

### Viés e avaliação de qualidade

O risco de viés foi avaliado utilizando a ferramenta Cochrane de Risco de Viés 2. Os domínios avaliados foram relato seletivo de desfechos, dados incompletos de desfechos, cegamento da avaliação de desfechos, cegamento de participantes e profissionais, geração de sequências: ocultação de alocação e outras questões. O risco de viés foi categorizado em baixo, alto ou incerto.

## Resultados

**Figura 1:**
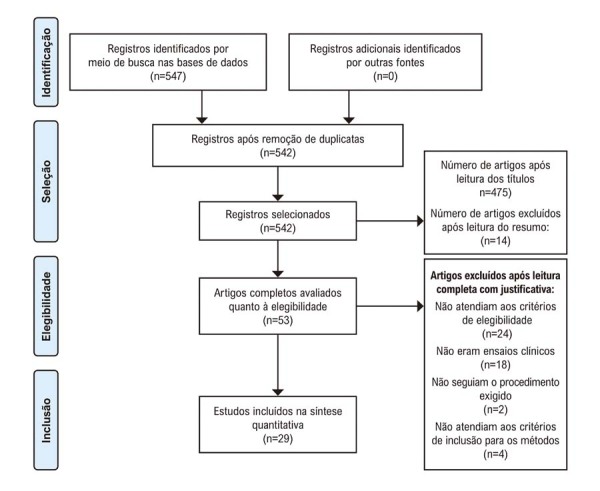
– Fluxograma da estratégia de pesquisa

De acordo com os dados apresentados no fluxograma de seleção de artigos, a busca inicial nas bases de dados resultou em um total de 547 artigos. Destes, 475 foram excluídos após a leitura dos títulos. Posteriormente, 72 artigos foram avaliados com base em seus resumos, e 14 foram considerados não diretamente relacionados ao tema deste estudo. Assim, 58 artigos foram selecionados para revisão de texto completo. Destes, 5 foram excluídos devido à duplicação, 18 não eram ensaios clínicos, 2 não seguiram o procedimento necessário e 4 não atenderam aos critérios de inclusão para os métodos. Portanto, esta revisão sistemática inclui 29 artigos que atenderam aos critérios de elegibilidade para inclusão ( Figura 1 ).

### Risco de viés

**Figura 2:**
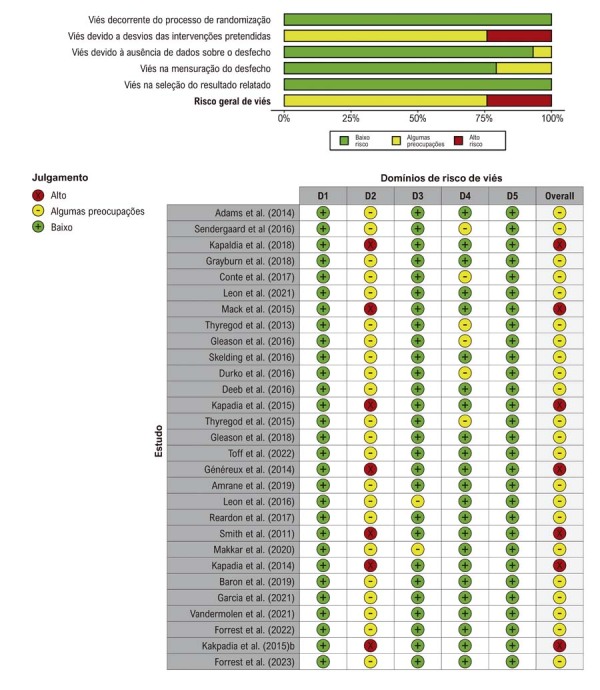
– Estratificação de risco de viés para todos os estudos incluídos usando a ferramenta Cochrane de Risco de Viés 2. Domínios - D1: Viés decorrente do processo de randomização; D2: Viés devido a desvios das intervenções pretendidas; D3: Viés devido à ausência de dados sobre o desfecho; D4: Viés na mensuração do desfecho; D5: Viés na seleção do resultado relatado.

O risco de viés foi avaliado em 8 ensaios ( Tabela 1 ). Cada ensaio foi classificado como “alto risco”, “baixo risco” ou “algumas preocupações” ( Figura 2 ).

**Tabela 1:** – Características basais de estudos selecionados

Estudo (Autor/ano)	País	Amostra	Desenho	Critérios de inclusão	Intervenções		Medidas	Resultados
					Intervenção	Controle		
Adams et al. 2014^10^	EUA	742 TAVI (n = 389) SAVR (n = 353)	Ensaio clínico randomizado	Pacientes com EA grave e sintomas de insuficiência cardíaca da New York Heart Association (NYHA) classe II	TAVI	Técnicas convencionais de SAVR de coração aberto com uso de bypass cardiopulmonar.	Mortalidade	Mortalidade Em 1 ano: 14,2% no grupo TAVI 19,1% no grupo SAVR Redução absoluta do risco de 4,9 pontos percentuais (intervalo de confiança, −0,4; P<0,001 para não inferioridade; P = 0,04 para superioridade).
Søndergaard et al. 2016^11^	Dinama	276 TAVI (n = 142) SAVR (n = 134)	Ensaio clínico randomizado	Estenose grave da valva aórtica nativa	TF-TAVI	SAVR	Mortalidade	Mortalidade Aos 2 anos 8,0% no grupo TAVI 9,8% no grupo SAVR p=0,54.
Skelding et al. 2016^12^	EUA	353 TAVI (n = 183) SAVR (n = 170)	Ensaio clínico randomizado	Pacientes do sexo feminino com sintomas de classe II ou superior da New York Heart Association (NYHA) e EA grave	TAVI	Cirurgia convencional SAVR	Mortalidade e Complicações Pós-Operatórias	Mortalidade em 30 dias Mortalidade por todas as causas 3,8% no grupo TAVI 4,7% no grupo SAVR p = 0,70 AVC: 5,6% no grupo TAVI 6,5% no grupo SAVR p = 0,73 Infarto do miocárdio: 1,6% no grupo TAVI 0,6% no grupo SAVR p = 0,35 Principais complicações vasculares 7,7% no grupo TAVI 0,6% no grupo SAVR p = 0,001 Perfuração cardíaca 2,7% no grupo TAVI 0,0% no grupo SAVR p = 0,03 Implante de marcapasso permanente 20,7% no grupo TAVI 7,2% no grupo SAVR p < 0,001
Grayburn et al. 2018^13^	EUA	739 TAVI (n = 386) SAVR (n = 353)	Ensaio clínico randomizado	Os pacientes foram estratificados pela gravidade da AR basal (regurgitação aórtica) naqueles com nenhum e/ou traços e aqueles com RA leve	TAVI	SAVR	Mortalidade	Mortalidade 49% (84 de 172 pacientes) no grupo TAVI 8% (13 de 162 pacientes) no grupo SAVR
Conte et al. 2017^14^	EUA	790 TAVI (n = 391) SARV (n = 359)	Ensaio clínico randomizado	EA grave	Portas de acesso iliofemoral ou não iliofemoral TAVI	SAVR	Complicações pós-operatórias, mortalidade	Mortalidade em 2 anos 8,0% no grupo TAVI 9,8% no grupo SAVR p = 0,54 Principais complicações vasculares: Significativamente maior no grupo TAVI durante o período periprocedimental (5,4% vs. 1,4%, p = 0,003)
Leon et al. 2021^15^	EUA	950 TAVI (n = 496) Cirurgia (n = 454)	Ensaio clínico randomizado	Pacientes com EA grave	TAVI transfemoral	Cirurgia minimamente invasiva	Mortalidade	Mortalidade em 2 anos TAVI: 57 pacientes (11,5%) SAVR: 78 pacientes (17,4%) p = 0,007
Durko et al. 2018 ^16^	Europa, EUA, Canadá	1660 TAVI (n = 864) Cirurgia (n = 796)	Ensaios clínicos randomizados	Pacientes com EA grave e sintomática com risco cirúrgico intermediário	TAVI	Cirurgia convencional SAVR	Complicações	AVC Precoce: TAVI: 3,3% SAVR: 5,4% p = 0,031 Encefalopatia precoce: TAVI: 1,6% SAVR: 7,8% p < 0,001
Thyregod et al. 2013^17^	Escandinávia	280 TAVI (n = 140) SAVR (n = 140)	Ensaio clínico randomizado	Pacientes com 70 anos ou mais com estenose VA degenerativa grave com sintomas ou sem sintomas, mas com disfunção sistólica e/ou hipertrofia ventricular esquerda	TAVI	Esternotomia mediana completa SAVR	Complicações ou mortalidade por todas as causas (desfecho primário)	Infarto do miocárdio, acidente vascular cerebral ou mortalidade por todas as causas. Grupo SAVR: 15% de incidência Grupo TAVI: 5% de incidência p<0,001
Gleason et al. 2016^18^	EUA	750 TAVI (n = 391) SAVR (n = 359)	Ensaio clínico randomizado	Pacientes de alto risco (mortalidade SAVR prevista de 15%) com EA grave	TAVI	SAVR	Mortalidade e complicações	Taxa de AVC em 30 dias: TAVI: 4,9% SAVR: 6,2% p = 0,46 Taxa de AVC em 1 ano: TAVI: 8,7% SAVR: 12,5% p = 0,11 Taxa de AVC em 2 anos: TAVI: 10,9% SAVR: 16,6% p = 0,05 Mortalidade por todas as causas em 2 anos: TAVI: 83,3% SAVR: 54,5% p = 0,29
Kapadia et al. 2018^19^	EUA	5637 TAVI (n = 4.389) SAVR (n = 1.248)	Ensaio clínico randomizado	Pacientes com EA	TF-TAVI	SAVR	Complicações e qualidade de vida	AVC em 30 dias: TF-TAVI: 5,1% SAVR: 3,7% p = 0,09 AVC grave em 30 dias: TF-TAVI: 3,9% SAVR: 2,2% p = 0,018 SAVR (Escore KCCQ): Sem acidente vascular cerebral grave: 79 [53, 94] Com acidente vascular cerebral grave: 64 [30, 94] p = 0,03 TF-TAVI (Escore KCCQ): Sem acidente vascular cerebral grave: 78 [49, 96] Com acidente vascular cerebral grave: 60 [8, 99] p = 0,04
Mack et al. 2015^20^	EUA	699 TAVI (n = 348) SAVR (n = 351)	Ensaio controlado randomizado	Pacientes com EA grave	TF-TAVI	Tipo de cirurgia SAVR não especificado	Mortalidade	Mortalidade Aos 5 anos 67,8% no grupo TAVI 62,4% no grupo SAVR p=0,76.
Deeb et al. 2016^21^	EUA	750 TAVI (n = 391) SAVR (n = 359)	Ensaio clínico randomizado	Pacientes com EA grave	TAVI	Procedimento SAVR a critério do cirurgião cardíaco	Mortalidade e complicações	Resultados de três anos Mortalidade por todas as causas ou acidente vascular cerebral 37,3% no grupo TAVI 46,7% no grupo SAVR p=0,006 Mortalidade por todas as causas: 32,9% no grupo TAVI; 39,1% no grupo SAVR; p=0,068 Todos os AVC 12,6% no grupo TAVI 19,0% no grupo SAVR p=0,034 Eventos cardiovasculares ou cerebrovasculares adversos graves: 40,2% no grupo TAVI; 47,9% no grupo SAVR; p=0,025
Kapadia et al. 2015^22^	EUA	358 TAVI (n = 179) SAVR (n = 179)	Ensaio clínico randomizado	Pacientes com EA grave sintomática inoperável (idade média de 83 anos, Sociedade de Cirurgiões Torácicos Risco previsto de mortalidade 11,7%, 54% mulheres).	TF-TAVI	Valvoplastia aórtica com balão	Mortalidade	Resultados de cinco anos Mortalidade por todas as causas 71,8% no grupo TAVI 93,6% no grupo SAVR p<0,0001
Thyregod et al. 2015^23^	Dinamarca e Suécia	274 TAVI (n = 139) SAVR (n = 135)	Ensaio clínico randomizado	Pacientes com idade ≥70 anos com estenose valvar aórtica degenerativa grave	TF-TAVI	SAVR foi submetido a cirurgia cardíaca aberta convencional com uso de bypass cardiopulmonar	Mortalidade e complicações	Resultados de um ano Morte por qualquer causa, acidente vascular cerebral ou infarto do miocárdio (desfecho primário) 13,1% no grupo TAVI 16,3% no grupo SAVR −3,2% de diferença absoluta; p=0,43 para superioridade
Gleason et al. 2018^24^	EUA	750 TAVI (n = 391) SAVR (n = 359)	Ensaio clínico randomizado	Pacientes com EA grave e sintomática (classe funcional II ou superior da New York Heart Association [NYHA])	TAVI	SAVR	Mortalidade e complicações	Resultados de cinco anos Mortalidade por todas as causas 55,3% no grupo TAVI 55,4% no grupo SAVR AVC grave 12,3% no grupo TAVI 13,2% no grupo SAVR
Toff et al. 2022^25^	Reino Unido	866 TAVI (n = 450) SAVR (n = 416)	Ensaio clínico randomizado	Os pacientes tinham 70 anos ou mais, EA grave e sintomática e risco operatório aumentado devido à comorbidade ou idade.	TAVI	SAVR (esternotomia mediana e cirurgia minimamente invasiva	Mortalidade	Resultados de um ano Mortalidade por todas as causas 4,6% no grupo TAVI 6,6% no grupo cirúrgico Diferença de risco absoluto ajustada −2,0% (IC unilateral de 97,5%, −∞ a 1,2%)
Généreux et al. 2014^26^	EUA	657 SARV (n = 313) TAVI-TF (n = 240) TARV-TA (n = 104)	Ensaio clínico randomizado	Pacientes com EA sintomática grave	TF-TAVI OU TA-TAVI	SAVR	Complicação pós-operatória	Resultados de 30 dias BC Grave (complicações sanguíneas) 22,7% após SAVR 11,3% após TF-TAVI 8,8% após TA-TAVI p<0,0001 Taxa de transfusão em 30 dias: 17,9% após SAVR; 7,1% após TF-TAVI 4,8% após TA-TAVIp<0,0001
Amrane et al. 2019^27^	Canadá, Europa, EUA	936 SARV (n = 438) TAVI (n = 498)	Ensaio clínico randomizado	Pacientes com EA sintomática grave são considerados de risco cirúrgico intermediário.	TF-TAVI	SAVR	Mortalidade	Resultados de um ano Mortalidade por todas as causas 6,5% após TAVI 6,7% após SAVR
Leon et al. 2016^28^	EUA	2032 TAVI (n = 1011) SAVR (n = 1021)	Ensaio clínico randomizado	Pacientes de risco intermediário com EA grave	TF-TAVI OU TA-TAVI	Acesso transtorácico ou transaórtico SAVR	Complicações pós-operatórias e mortalidade	Morte por qualquer causa ou acidente vascular cerebral incapacitante Geral: taxas semelhantes entre TAVI e cirurgia p=0,001 para não inferioridade
Reardon et al. 2011^29^	EUA, Canadá, Europa	1657 TAVI (n = 863) SAVR (n = 794)	Ensaio clínico randomizado	Pacientes de risco intermediário com EA sintomática grave	TF-TAVI	SAVR	Complicações pós-operatórias, qualidade de vida e mortalidade	Resultados de 24 meses Mortalidade por todas as causas 11,4% no grupo TAVI 11,6% no grupo cirúrgico Intervalo de confiança de 95% para diferença, −3,8% a 3,3% Acidente vascular cerebral incapacitante Taxas semelhantes em ambos os grupos Qualidade de vida (Escore resumide do KCCQ) Melhora significativa em ambos os grupos ao longo de 24 meses Uma maior proporção de pacientes melhorou em 1 mês no grupo TAVI em comparação ao grupo cirúrgico
Smith et al. 2011^30^	EUA, Canadá, Alemanha	699 SARV (n = 351) TAVI-TF (n = 244) TARV-TA (n = 104)	Ensaio clínico randomizado	Pacientes com EA grave e sintomas cardíacos (New York Heart Association [NYHA] função de classe II ou pior)	TF-TAVI OU TA-TAVI	Cirurgia convencional SAVR	Complicações pós-operatórias e mortalidade	Resultados de 30 dias e 1 ano Morte por todas as causas 30 dias: 3,4% no grupo transcateter vs. 6,5% no grupo cirúrgico (p=0,07) 1 ano: 24,2% no grupo transcateter vs. 26,8% no grupo cirúrgico (p=0,44) Redução de 2,6 pontos percentuais no grupo transcateter Limite superior do IC de 95% para diferença: 3,0 pontos percentuais Margem predefinida: 7,5 pontos percentuais; p=0,001 para não inferioridade AVC grave 30 dias: 3,8% no grupo transcateter vs. 2,1% no grupo cirúrgico (p=0,20) 1 ano: 5,1% no grupo transcateter vs. 2,4% no grupo cirúrgico (p=0,07) Principais complicações vasculares 30 dias: 11,0% no grupo transcateter vs. 3,2% no grupo cirúrgico (p<0,001)
Makkar et al. 2020^31^	EUA	2032 TARV-TF (n = 1550) SAVR (n = 482)	Ensaio clínico randomizado	Pacientes de risco intermediário com EA sintomática grave	TF-TAVI	SAVR na região transtorácica	Mortalidade, Complicações pós-operatórias, Qualidade de vida	Resultados de cinco anos Mortalidade por todas as causas 47,9% no grupo TAVI 43,4% no grupo cirúrgico p=0,21 Pelo menos regurgitação aórtica paravalvar leve 33,3% no grupo TAVI 6,3% no grupo cirúrgico Melhoria do estado de saúde Melhora semelhante nos grupos TAVI e cirúrgico em 5 anos
Kapadia et al. 2014^32^	EUA	449 TAVI (n = 220) SAVR (n = 229)	Ensaio clínico randomizado	Pacientes inoperáveis com EA grave	TAVI TF	SAVR TF	Mortalidade	Mortalidade em três anos 54,1% no grupo TAVI 80,9% no grupo SAVR p<0,001
Baron et al. 2019^33^	EUA	943 TAVI (n = 494) SAVR (n = 449)	Ensaio clínico randomizado	Pacientes com EA grave de baixo risco cirúrgico	TAVI transfemoral usando uma válvula expansível por balão	SAVR	Qualidade de vida	Em 1 mês TAVI apresentou melhor estado de saúde que SAVR Diferença média no KCCQ-OS: 16,0 pontos, p<0,001 Aos 6 meses TAVI permaneceu melhor que SAVR, embora com efeito reduzido Diferença média no KCCQ-OS: 2,6 pontos, p<0,04 Aos 12 meses TAVI ainda apresentou melhor estado de saúde, com efeito ainda mais reduzido Diferença média no KCCQ-OS: 1,8 pontos, p<0,04 Proporção de pacientes com KCCQ-OS ≥75 e sem declínio significativo em relação ao valor basal Aos 6 meses: 90,3% em TAVI vs. 85,3% em SAVR, p=0,03 Aos 12 meses: 87,3% em TAVI vs. 82,8% em SAVR, p=0,07
Garcia et al. 2021^34^	EUA	1524 TAVI (n = 1045) SAVR (n = 479)	Ensaio clínico randomizado	Pacientes com EA e doença renal crônica (DRC) submetidos a TAVI ou SAVR	TAVI	SAVR	Complicação	Lesão Renal Aguda Perioperatória (LRA) 26,3% após SAVR 10,3% após TAVI p<0,001
Vandermolen et al. 2021^35^	EUA	322 TAVI (n = 220) SAVR (n = 102)	Ensaio clínico randomizado	Pacientes com EA clássica de baixo fluxo e baixo gradiente	TAVI	SAVR	Mortalidade	Acompanhamento mediano de 2,7 anos (intervalo de 1,5 a 4,1 anos) 99 pacientes morreram: 70 (31,8%) no grupo TAVI 29 (28,4%) no grupo SAVR
Forrest et al. 2022^36^	EUA	1414 TAVI (n = 730) SAVR (n = 684)	Ensaio clínico randomizado	Pacientes com EA em pacientes de baixo risco	TAVI	SAVR	Mortalidade e complicações	Taxas de mortalidade por todas as causas 3,5% no grupo TAVI 4,4% no grupo SAVR p=0,366 Taxas de acidente vascular cerebral incapacitantes 1,5% no grupo TAVI 2,7% no grupo SAVR p=0,119
Kapadia et al. 2015^23^	EUA	358 TAVI (n = 179) SAVR (n = 179)	Ensaio clínico randomizado	Pacientes com EA grave inoperável	TAVI	SAVR	Mortalidade	Resultados de cinco anos Mortalidade por todas as causas 71,8% no grupo TAVI 93,6% no grupo SAVR p<0,0001
Forrest et al. 2023^37^	EUA	1414 TAVI (n = 730) SAVR (n = 684)	Ensaio clínico randomizado	Pacientes de baixo risco com EA	TAVI com válvula supraanular autoexpansível	SAVR	Mortalidade e complicações	Taxas de mortalidade (AVC por todas as causas ou incapacitante) Ano 1: −1,8% de diferença (TAVI vs. Cirurgia) Ano 2: −2,0% de diferença Ano 3: −2,9% de diferença Incidência de regurgitação paravalvar leve 20,3% no grupo TAVI 2,5% no grupo cirúrgico Colocação de marcapasso 23,2% no grupo TAVI 9,1% no grupo cirúrgico p<0,001 Regurgitação paravalvar moderada ou maior <1% em ambos os grupos Nenhuma diferença significativa

TF-TAVI: implante transcateter de válvula aórtica transfemoral; SAVR: substituição cirúrgica de válvula aórtica; AR: regurgitação aórtica; NYHA: Classificação Funcional da New York Heart Association; TA-TAVI: implante transcateter de válvula aórtica transfemoral; LDB: hemorragia com risco de vida ou incapacitante; LRA: lesão renal aguda; VA: válvula aórtica; KCCQ: Questionário de Cardiomiopatia de Kansas City; KCCQ-OS: Escore Geral Resumido do Questionário de Cardiomiopatia de Kansas City; HR: razão de risco; IC: intervalo de confiança; EUA: Estados Unidos da América; RU: Reino Unido; EA: estenose aórtica.

### Participantes

Um total de 19.567 pacientes recebeu a intervenção nos estudos incluídos nesta revisão. A idade variou de 73 a 83 anos, e a prevalência foi do sexo masculino (58,5%). Os critérios de inclusão foram pacientes com disfunção aórtica submetidos ao procedimento TAVI ou SAVR. Os demais dados são apresentados na Tabela 1 . A Figura Central apresenta um resumo dos achados relacionados aos artigos selecionados.^10 - 37^

### Intervenção

Dos estudos incluídos nesta revisão, a CoreValve^9 , 17 , 20 , 22^ foi a válvula mais comumente utilizada para TAVI, sendo empregada na maioria dos procedimentos. No entanto, outras válvulas também foram relatadas, incluindo a Edwards SAPIEN 1ª geração,^21^ a válvula cardíaca expansível por balão SAPIEN XT^15 , 20 , 27^e a SAPIEN XT 2ª geração, todas feitas de pericárdio bovino ou suíno,^18 , 19^ bem como a válvula Sapien 3.^14^ A via de acesso preferencial para TAVI foi a transfemoral; no entanto, em alguns estudos, o acesso transapical também foi utilizado, embora em menor extensão.

## Discussão

Esta revisão sistemática foi iniciada com o objetivo de avaliar o impacto do TAVI nos desfechos clínicos, com ênfase na mortalidade, hospitalização, complicações pós-operatórias e qualidade de vida. Nesse sentido, uma revisão dos artigos estudados mostrou que as taxas de mortalidade foram significativamente menores^14 , 20 , 21 , 31 , 24 , 36^em pacientes submetidos ao TAVI, juntamente com melhores perspectivas em termos de qualidade de vida.^18^ Além disso, o estudo revelou que a taxa de complicações pós-operatórias, como sangramento e lesão renal aguda, foi menor no grupo TAVI. É importante esclarecer que a subanálise dos estudos incluídos não faz parte da análise, de modo que o mesmo paciente pode ser contado mais de uma vez. Portanto, a duplicação de pacientes na contagem é possível. Outro fator que deve ser levado em consideração é a idade. Todos os estudos selecionados investigaram indivíduos com mais de 70 anos de idade.

Como mostrado, a maioria dos artigos demonstra que o TAVI tem melhor desempenho do que o SAVR em termos de mortalidade. Adams et al.^10^ sugerem que vários fatores podem ter contribuído para o desempenho superior do TAVI, como a abordagem menos invasiva, mobilização mais rápida e recuperação pós-operatória, juntamente com menores taxas de acidentes vasculares cerebrais e outras complicações vasculares. Além disso, os autores relataram que uma maior taxa de regurgitação paravalvar, comum em pacientes com TAVI, não impactou a sobrevida global em seu estudo. Há outros artigos, como o de Mack et al.,^20^ que argumentam que a taxa substancialmente maior de regurgitação paravalvar no TAVI resulta em menor taxa de sobrevida para os pacientes. No entanto, isso não é considerado um fator que comprometa o TAVI como uma alternativa à cirurgia convencional.

Em relação à incidência de complicações pós-operatórias^13 , 14^os artigos mostraram pouca diferença entre TAVI e SAVR. No entanto, o estudo de Durko et al.^16^ revelou um achado notável: a maior incidência de encefalopatia após SAVR em comparação com TAVI. Isso pode ser atribuído ao uso de circulação extracorpórea na SAVR, que leva à redução da oxigenação do cérebro, aumentando o risco de encefalopatia. Além disso, há uma menor taxa de AVC precoce na TAVI em comparação com SAVR, o que está relacionado ao menor risco cirúrgico, aos avanços tecnológicos no procedimento e à técnica de implante utilizada.

Além disso, o estudo de Généreux et al.^26^ mostrou que complicações hemorrágicas e transfusões foram 2 a 3 vezes mais frequentes no grupo SAVR em comparação ao TAVI, o que o autor não considera surpreendente, dada a natureza mais invasiva da cirurgia e a coagulopatia bem documentada que ocorre após o bypass cardiopulmonar, o que respalda sua afirmação.

O estudo de Reardon et al.,^29^ por exemplo, mostrou que a SAVR estava associada a maiores taxas de lesão renal aguda, fibrilação atrial e necessidade de transfusão. A natureza invasiva do procedimento pode explicar parcialmente essas complicações, visto que cirurgias de grande porte tendem a prolongar a internação hospitalar, o tempo de ventilação mecânica e a necessidade de medicamentos inotrópicos e suporte circulatório mecânico. No entanto, é importante reconhecer que fatores específicos do paciente e técnicas cirúrgicas também podem influenciar esses desfechos, e estudos adicionais relataram resultados variados em relação à incidência de tais complicações. Por outro lado, o TAVI demonstrou maiores taxas de regurgitação aórtica residual e necessidade de implante de marcapasso. Esses problemas podem advir de trauma, isquemia, hemorragia e edema ao nível do nó atrioventricular, bem como da própria natureza da válvula, considerando as diferenças entre válvulas expansíveis por balão e autoexpansíveis. Notavelmente, a incidência de implante de marcapasso e regurgitação paravalvar evoluiu ao longo do tempo com avanços tecnológicos e técnicas de implante aprimoradas. Esses achados sugerem uma melhor qualidade de vida associada ao TAVI nos primeiros 30 dias em comparação ao SAVR. No entanto, após seis meses, a qualidade de vida parece ser comparável entre os dois procedimentos, reforçando a necessidade de acompanhamento de longo prazo para avaliar a durabilidade e os resultados clínicos.

Corroborando a ideia mencionada acima, o estudo de Baron et al.^33^ também destaca que o TAVI demonstra resultados superiores em termos de qualidade de vida em comparação à SAVR, um achado consistente com o de Reardon et al.^29^ Baron et al.^33^ atribuem isso às maiores taxas de sangramento, lesão renal aguda e fibrilação atrial pós-operatória na SAVR, bem como às taxas relativamente menores de lesão vascular e vazamento paravalvar no TAVI. Esses fatores são considerados essenciais para a melhor qualidade de vida observada em pacientes submetidos ao TAVI. Essa explicação ajuda a justificar por que o escore do KCCQ é melhor em pacientes submetidos ao TAVI até 6 meses, embora essa diferença diminua substancialmente ao longo do tempo.

A comparação entre a substituição valvar aórtica transcateter e a substituição valvar aórtica cirúrgica convencional tem sido um tópico central em vários estudos randomizados, especialmente em pacientes com EA grave. Os dados apresentados por Adams et al.^10^ mostram uma vantagem significativa do TAVI em termos de mortalidade, com uma redução absoluta de 4,9 pontos percentuais na taxa de mortalidade em comparação com a SAVR após 1 ano, com p = 0,04 para superioridade. Isso se alinha com os achados de outros estudos, como Thyregod et al.,^17^ que também demonstraram a superioridade do TAVI em termos de complicações graves e mortalidade. Esses resultados sugerem que, para certos grupos de pacientes, como aqueles com alto risco cirúrgico ou condições comórbidas, o TAVI pode ser uma alternativa mais segura e eficaz, especialmente quando se considera a recuperação pós-operatória e o tempo reduzido de hospitalização.

Ao longo do tempo, o TAVI evoluiu significativamente desde sua introdução há mais de duas décadas, com melhorias nos designs das válvulas, nas técnicas de procedimento e nos critérios de seleção de pacientes. Benefícios a curto prazo, incluindo menores taxas de mortalidade e complicações, foram bem documentados. No entanto, dados de longo prazo sugerem a necessidade de mais pesquisas para avaliar a durabilidade das válvulas, as complicações tardias e os resultados funcionais após cinco anos.

Uma análise evolutiva das próteses para TAVI pode ser altamente valiosa, visto que melhorias significativas foram feitas ao longo do tempo, particularmente na redução do vazamento paravalvar. Avanços no design, nos materiais e nas técnicas de aplicação das válvulas levaram a uma melhor vedação e a um posicionamento mais preciso das válvulas, contribuindo para melhores resultados clínicos e durabilidade a longo prazo. Esses aprimoramentos ajudaram a abordar um dos principais desafios do TAVI, permitindo melhor desempenho hemodinâmico e menor incidência de complicações, o que, em última análise, beneficia os pacientes submetidos a esse procedimento menos invasivo.

O estudo apresenta algumas limitações que podem impactar a interpretação dos resultados. A inclusão de diferentes tipos e marcas de válvulas pode introduzir variabilidade, dificultando a comparação direta dos efeitos de cada modelo. Além disso, diferenças nas técnicas cirúrgicas e protocolos entre as equipes médicas envolvidas nos ensaios podem afetar a consistência dos resultados, limitando a aplicabilidade dos achados a práticas clínicas mais amplas. A heterogeneidade nas populações de pacientes e nos cenários clínicos também pode introduzir viés, dificultando a generalização dos resultados para todos os contextos. Além disso, os critérios de inclusão utilizados podem ter excluído dados relevantes, reduzindo a representatividade do estudo para populações mais diversas.

Apesar dessas limitações, os resultados do estudo fornecem insights valiosos para a prática clínica. As diferentes abordagens e tipos de válvulas analisadas oferecem uma compreensão mais ampla das opções disponíveis, o que pode ser benéfico na personalização de tratamentos e na otimização de resultados para diferentes perfis de pacientes. A abordagem TAVI, por exemplo, demonstrou benefícios claros, particularmente com a técnica transfemoral, que pode estar associada a melhor recuperação e menor risco cirúrgico. Mesmo com a necessidade de cautela na generalização dos resultados, os achados contribuem significativamente para o avanço da compreensão em intervenções cardíacas, fornecendo uma base sólida para futuras investigações e melhorias nas práticas clínicas.

## Conclusão

O TAVI em pacientes com EA reduziu a mortalidade em comparação com a SAVR. O TAVI parece reduzir o tempo de internação hospitalar, o sangramento e a lesão renal aguda, além de melhorar a qualidade de vida. Deve-se considerar, com base nos artigos selecionados, que os idosos (acima de 70 anos) são a população mais exposta a esse tipo de intervenção, provavelmente devido ao alto risco cirúrgico de um procedimento aberto.

## Supplement

Search strategy in PubMed

## Material Suplementar

*Material suplementar Para informação adicional, por favor, clique aqui.
