# Supplementary material for: Implante de Válvula Aórtica Transcateter (TAVI) versus Substituição Cirúrgica de Válvula em Resultados Clínicos em Pacientes com Estenose Aórtica
Source: Arq Bras Cardiol. 2025 Oct 28;122(10):e20240295. [Article in Portuguese] doi: 10.36660/abc.20240295 (PMC12677903; doi:10.36660/abc.20240295)
Supplement: Search strategy in PubMed [file 0066-782x-abc-122-10-e20240295-suppl01.pdf]

Supplement

Search strategy in PubMed

("aortic valve stenosis"[MeSH Terms] OR ("aortic"[All Fields] AND "valve"[All Fields] AND "stenosis"[All Fields]) OR "aortic valve stenosis"[All Fields] OR ("aortic"[All Fields] AND "stenosis"[All Fields]) OR "aortic stenosis"[All Fields] OR ("aortic valve stenosis"[MeSH Terms] OR ("aortic"[All Fields] AND "valve"[All Fields] AND "stenosis"[All Fields]) OR "aortic valve stenosis"[All Fields] OR ("aortic"[All Fields] AND "valve"[All Fields] AND "stenoses"[All Fields]) OR "aortic valve stenoses"[All Fields]) OR ("aortic valve stenosis"[MeSH Terms] OR ("aortic"[All Fields] AND "valve"[All Fields] AND "stenosis"[All Fields]) OR "aortic valve stenosis"[All Fields] OR ("stenoses"[All Fields] AND "aortic"[All Fields]) OR "stenoses aortic"[All Fields]) OR ("aortic valve stenosis"[MeSH Terms] OR ("aortic"[All Fields] AND "valve"[All Fields] AND "stenosis"[All Fields]) OR "aortic valve stenosis"[All Fields] OR ("stenoses"[All Fields] AND "aortic"[All Fields] AND "valve"[All Fields]) OR "stenoses aortic valve"[All Fields]) OR ("aortic valve stenosis"[MeSH Terms] OR ("aortic"[All Fields] AND "valve"[All Fields] AND "stenosis"[All Fields]) OR "aortic valve stenosis"[All Fields] OR ("stenosis"[All Fields] AND "aortic"[All Fields]) OR "stenosis aortic"[All Fields]) OR ("aortic valve stenosis"[MeSH Terms] OR ("aortic"[All Fields] AND "valve"[All Fields] AND "stenosis"[All Fields]) OR "aortic valve stenosis"[All Fields] OR ("stenosis"[All Fields] AND "aortic"[All Fields] AND "valve"[All Fields]) OR "stenosis aortic valve"[All Fields]) OR ("aortic valve stenosis"[MeSH Terms] OR ("aortic"[All Fields] AND "valve"[All Fields] AND "stenosis"[All Fields]) OR "aortic valve stenosis"[All Fields] OR ("valve"[All Fields] AND "stenoses"[All Fields] AND "aortic"[All Fields]) OR "valve stenoses aortic"[All Fields]) OR ("aortic valve stenosis"[MeSH Terms] OR ("aortic"[All Fields] AND "valve"[All Fields] AND "stenosis"[All Fields]) OR "aortic valve stenosis"[All Fields] OR ("valve"[All Fields] AND "stenosis"[All Fields] AND "aortic"[All Fields]) OR "valve stenosis aortic"[All Fields]) OR ("aortic valve stenosis"[MeSH Terms] OR ("aortic"[All Fields] AND "valve"[All Fields] AND "stenosis"[All Fields]) OR "aortic valve stenosis"[All Fields])) AND ("transcatheter aortic valve replacement"[MeSH Terms] OR ("transcatheter"[All Fields] AND "aortic"[All Fields] AND "valve"[All Fields] AND "replacement"[All Fields]) OR "transcatheter aortic valve replacement"[All Fields] OR ("transcatheter"[All Fields] AND "aortic"[All Fields] AND "valve"[All Fields] AND "implantation"[All

Fields]) OR "transcatheter aortic valve implantation"[All Fields] OR ("transcatheter aortic valve replacement"[MeSH Terms] OR ("transcatheter"[All Fields] AND "aortic"[All Fields] AND "valve"[All Fields] AND "replacement"[All Fields]) OR "transcatheter aortic valve replacement"[All Fields])) AND ((("conventional"[All Fields] OR "conventionals"[All Fields]) AND ("therapeutics"[MeSH Terms] OR "therapeutics"[All Fields] OR "treatments"[All Fields] OR "therapy"[MeSH Subheading] OR "therapy"[All Fields] OR "treatment"[All Fields] OR "treatment s"[All Fields])) OR ("sternotomy"[MeSH Terms] OR "sternotomy"[All Fields] OR "sternotomies"[All Fields]) OR ("thoracotomy"[MeSH Terms] OR "thoracotomy"[All Fields] OR "thoracotomies"[All Fields])) AND ("hospital s"[All Fields] OR "hospitalisation"[All Fields] OR "hospitalization"[MeSH Terms] OR "hospitalization"[All Fields] OR "hospitalised"[All Fields] OR "hospitalising"[All Fields] OR "hospitality"[All Fields] OR "hospitalisations"[All Fields] OR "hospitalizations"[All Fields] OR "hospitalize"[All Fields] OR "hospitalized"[All Fields] OR "hospitalizing"[All Fields] OR "hospitals"[MeSH Terms] OR "hospitals"[All Fields] OR "hospital"[All Fields] OR ("length of stay"[MeSH Terms] OR ("length"[All Fields] AND "stay"[All Fields]) OR "length of stay"[All Fields] OR ("hospital"[All Fields] AND "stay"[All Fields]) OR "hospital stay"[All Fields]) OR ("length of stay"[MeSH Terms] OR ("length"[All Fields] AND "stay"[All Fields]) OR "length of stay"[All Fields] OR ("hospital"[All Fields] AND "stays"[All Fields]) OR "hospital stays"[All Fields]) OR ("length of stay"[MeSH Terms] OR ("length"[All Fields] AND "stay"[All Fields]) OR "length of stay"[All Fields] OR ("stay"[All Fields] AND "length"[All Fields]) OR "stay length"[All Fields]) OR ("length of stay"[MeSH Terms] OR ("length"[All Fields] AND "stay"[All Fields]) OR "length of stay"[All Fields] OR ("stay"[All Fields] AND "lengths"[All Fields]) OR "stay lengths"[All Fields]) OR ("length of stay"[MeSH Terms] OR ("length"[All Fields] AND "stay"[All Fields]) OR "length of stay"[All Fields] OR ("stay"[All Fields] AND "hospital"[All Fields]) OR "stay hospital"[All Fields]) OR ("length of stay"[MeSH Terms] OR ("length"[All Fields] AND "stay"[All Fields]) OR "length of stay"[All Fields] OR ("stays"[All Fields] AND "hospital"[All Fields]) OR "stays hospital"[All Fields]) OR ("length of stay"[MeSH Terms] OR ("length"[All Fields] AND "stay"[All Fields]) OR "length of stay"[All Fields]) OR ("intensive care units"[MeSH Terms] OR ("intensive"[All Fields] AND "Care"[All Fields] AND "units"[All Fields]) OR "intensive care units"[All Fields]) OR ("intensive"[All Fields] OR "intensives"[All Fields]) OR ("Care"[All Fields] AND "Unit"[All Fields] AND ("intensive"[All Fields] OR

"intensives"[All Fields])) OR ("Care"[All Fields] AND ("unit s"[All Fields] OR "units"[All Fields]) AND ("intensive"[All Fields] OR "intensives"[All Fields])) OR ("intensive care units"[MeSH Terms] OR ("intensive"[All Fields] AND "Care"[All Fields] AND "units"[All Fields]) OR "intensive care units"[All Fields] OR ("icu"[All Fields] AND "intensive"[All Fields] AND "Care"[All Fields] AND "units"[All Fields]) OR "icu intensive care units"[All Fields]) OR ("intensive care units"[MeSH Terms] OR ("intensive"[All Fields] AND "Care"[All Fields] AND "units"[All Fields]) OR "intensive care units"[All Fields] OR ("Unit"[All Fields] AND "intensive"[All Fields] AND "Care"[All Fields]) OR "unit intensive care"[All Fields]) OR (("unit s"[All Fields] OR "units"[All Fields]) AND ("critical care"[MeSH Terms] OR ("critical"[All Fields] AND "Care"[All Fields]) OR "critical care"[All Fields] OR ("intensive"[All Fields] AND "Care"[All Fields]) OR "intensive care"[All Fields])) OR ("quality of life"[MeSH Terms] OR ("quality"[All Fields] AND "life"[All Fields]) OR "quality of life"[All Fields]) OR ("quality of life"[MeSH Terms] OR ("quality"[All Fields] AND "life"[All Fields]) OR "quality of life"[All Fields] OR ("health"[All Fields] AND "related"[All Fields] AND "quality"[All Fields] AND "life"[All Fields]) OR "health related quality of life"[All Fields]) OR ("quality of life"[MeSH Terms] OR ("quality"[All Fields] AND "life"[All Fields]) OR "quality of life"[All Fields] OR ("health"[All Fields] AND "related"[All Fields] AND "quality"[All Fields] AND "life"[All Fields]) OR "health related quality of life"[All Fields]) OR ("hrqols"[All Fields] OR "quality of life"[MeSH Terms] OR ("quality"[All Fields] AND "life"[All Fields]) OR "quality of life"[All Fields] OR "hrqol"[All Fields]) OR ("quality of life"[MeSH Terms] OR ("quality"[All Fields] AND "life"[All Fields]) OR "quality of life"[All Fields]) OR ("age specific"[All Fields] AND ("mortality"[MeSH Terms] OR "mortality"[All Fields] OR ("mortality"[All Fields] AND "rate"[All Fields]) OR "mortality rate"[All Fields])) OR ("age specific"[All Fields] AND ("mortality"[MeSH Terms] OR "mortality"[All Fields] OR ("mortality"[All Fields] AND "rate"[All Fields]) OR "mortality rate"[All Fields])) OR ("age specific"[All Fields] AND ("mortality"[MeSH Terms] OR "mortality"[All Fields] OR ("mortality"[All Fields] AND "rate"[All Fields]) OR "mortality rate"[All Fields])) OR ("mortality"[MeSH Terms] OR "mortality"[All Fields] OR ("case"[All Fields] AND "fatality"[All Fields] AND "rate"[All Fields]) OR "case fatality rate"[All Fields]) OR ("mortality"[MeSH Terms] OR "mortality"[All Fields] OR ("case"[All Fields] AND "fatality"[All Fields] AND "rate"[All Fields]) OR "case fatality rate"[All Fields]) OR (("mortality"[MeSH Terms] OR "mortality"[All Fields] OR ("case"[All Fields] AND "fatality"[All Fields] AND

"rate"[All Fields]) OR "case fatality rate"[All Fields]) AND ("card fail rev"[Journal] OR "cfr"[All Fields])) OR ("mortality"[MeSH Terms] OR "mortality"[All Fields] OR ("crude"[All Fields] AND "mortality"[All Fields] AND "rate"[All Fields]) OR "crude mortality rate"[All Fields]) OR ("mortality"[MeSH Terms] OR "mortality"[All Fields] OR ("crude"[All Fields] AND "mortality"[All Fields] AND "rate"[All Fields]) OR "crude mortality rate"[All Fields]) OR ("mortality"[MeSH Terms] OR "mortality"[All Fields] OR ("crude"[All Fields] AND "mortality"[All Fields] AND "rate"[All Fields]) OR "crude mortality rate"[All Fields]) OR ("mortality"[MeSH Terms] OR "mortality"[All Fields] OR ("crude"[All Fields] AND "mortality"[All Fields] AND "rate"[All Fields]) OR "crude mortality rate"[All Fields]) OR ("age specific"[All Fields] AND ("mortality"[MeSH Terms] OR "mortality"[All Fields] OR ("mortality"[All Fields] AND "rate"[All Fields]) OR "mortality rate"[All Fields])) OR ("mortality"[MeSH Terms] OR "mortality"[All Fields] OR ("crude"[All Fields] AND "mortality"[All Fields] AND "rate"[All Fields]) OR "crude mortality rate"[All Fields]) OR ("mortality"[MeSH Terms] OR "mortality"[All Fields] OR ("mortality"[All Fields] AND "rate"[All Fields]) OR "mortality rate"[All Fields]) OR (("mortality"[MeSH Terms] OR "mortality"[All Fields] OR ("mortality"[All Fields] AND "Rates"[All Fields]) OR "mortality rates"[All Fields]) AND "age specific"[All Fields]) OR ("mortality"[MeSH Terms] OR "mortality"[All Fields] OR ("decline"[All Fields] AND "mortality"[All Fields]) OR "decline mortality"[All Fields]) OR ("mortality"[MeSH Terms] OR "mortality"[All Fields] OR ("decline"[All Fields] AND "mortality"[All Fields]) OR "decline mortality"[All Fields]) OR ("mortality"[MeSH Terms] OR "mortality"[All Fields] OR ("determinant"[All Fields] AND "mortality"[All Fields]) OR "determinant mortality"[All Fields]) OR ("mortality"[MeSH Terms] OR "mortality"[All Fields] OR ("determinant"[All Fields] AND "mortality"[All Fields]) OR "determinant mortality"[All Fields]) OR ("mortality"[MeSH Terms] OR "mortality"[All Fields] OR ("determinant"[All Fields] AND "mortality"[All Fields]) OR "determinant mortality"[All Fields]) OR (("mortality"[MeSH Terms] OR "mortality"[All Fields] OR "mortalities"[All Fields] OR "mortality"[MeSH Subheading]) AND ("cell differentiation"[MeSH Terms] OR ("cell"[All Fields] AND "differentiation"[All Fields]) OR "cell differentiation"[All Fields] OR "differentiated"[All Fields] OR "differentiation"[All Fields] OR "differential"[All Fields] OR "differentials"[All Fields] OR "differentiate"[All Fields] OR "differentiates"[All Fields] OR "differentiating"[All Fields] OR "differentiational"[All Fields] OR "differentiations"[All Fields] OR "differentiative"[All

Fields])) OR (("mortality"[MeSH Terms] OR "mortality"[All Fields] OR "mortalities"[All Fields] OR "mortality"[MeSH Subheading]) AND ("excess"[All Fields] OR "excesses"[All Fields] OR "excessive"[All Fields] OR "excessively"[All Fields])) OR (("mortality"[MeSH Terms] OR "mortality"[All Fields] OR "mortalities"[All Fields] OR "mortality"[MeSH Subheading]) AND ("excess"[All Fields] OR "excesses"[All Fields] OR "excessive"[All Fields] OR "excessively"[All Fields])) OR ("mortality"[MeSH Terms] OR "mortality"[All Fields] OR "mortalities"[All Fields] OR "mortality"[MeSH Subheading]) OR (("mortality"[MeSH Terms] OR "mortality"[All Fields] OR "mortalities"[All Fields] OR "mortality"[MeSH Subheading]) AND ("mortality"[MeSH Terms] OR "mortality"[All Fields] OR "mortalities"[All Fields] OR "mortality"[MeSH Subheading]) AND ("cell differentiation"[MeSH Terms] OR ("cell"[All Fields] AND "differentiation"[All Fields]) OR "cell differentiation"[All Fields] OR "differentiated"[All Fields] OR "differentiation"[All Fields] OR "differential"[All Fields] OR "differentials"[All Fields] OR "differentiate"[All Fields] OR "differentiates"[All Fields] OR "differentiating"[All Fields] OR "differentiational"[All Fields] OR "differentiations"[All Fields] OR "differentiative"[All Fields])) OR (("mortality"[MeSH Terms] OR "mortality"[All Fields] OR "mortalities"[All Fields] OR "mortality"[MeSH Subheading]) AND ("mortality"[MeSH Terms] OR "mortality"[All Fields] OR "mortalities"[All Fields] OR "mortality"[MeSH Subheading]) AND ("excess"[All Fields] OR "excesses"[All Fields] OR "excessive"[All Fields] OR "excessively"[All Fields])) OR ("decline"[All Fields] OR "declined"[All Fields] OR "decliner"[All Fields] OR "decliners"[All Fields] OR "declines"[All Fields] OR "declining"[All Fields]) OR ("mortality"[MeSH Terms] OR "mortality"[All Fields] OR ("mortality"[All Fields] AND "decline"[All Fields]) OR "mortality decline"[All Fields]) OR ("mortality"[MeSH Terms] OR "mortality"[All Fields] OR ("mortality"[All Fields] AND "determinant"[All Fields]) OR "mortality determinant"[All Fields]) OR ("mortality"[MeSH Terms] OR "mortality"[All Fields] OR ("mortality"[All Fields] AND "determinant"[All Fields]) OR "mortality determinant"[All Fields]) OR ("mortality"[MeSH Terms] OR "mortality"[All Fields] OR ("mortality"[All Fields] AND "rate"[All Fields]) OR "mortality rate"[All Fields]) OR ("mortality"[MeSH Terms] OR "mortality"[All Fields] OR ("mortality"[All Fields] AND "rate"[All Fields] AND "crude"[All Fields]) OR "mortality rate crude"[All Fields]) OR ("mortality"[MeSH Terms] OR "mortality"[All Fields] OR "mortalities"[All Fields] OR "mortality"[MeSH Subheading]) OR (("mortality"[MeSH Terms] OR "mortality"[All Fields] OR

("mortality"[All Fields] AND "Rates"[All Fields]) OR "mortality rates"[All Fields]) AND ("cell differentiation"[MeSH Terms] OR ("cell"[All Fields] AND "differentiation"[All Fields]) OR "cell differentiation"[All Fields] OR "differentiated"[All Fields] OR "differentiation"[All Fields] OR "differential"[All Fields] OR "differentials"[All Fields] OR "differentiate"[All Fields] OR "differentiates"[All Fields] OR "differentiating"[All Fields] OR "differentiational"[All Fields] OR "differentiations"[All Fields] OR "differentiative"[All Fields])) OR ("mortality"[MeSH Terms] OR "mortality"[All Fields] OR ("mortality"[All Fields] AND "excess"[All Fields]) OR "mortality excess"[All Fields]) OR (("j rehabil assist technol eng"[Journal] OR "rate"[All Fields]) AND ("agrosyst geosci environ"[Journal] OR "age"[Journal] OR "age omaha"[Journal] OR "age dordr"[Journal] OR "adv genet eng"[Journal] OR "age"[All Fields]) AND ("sensitivity and specificity"[MeSH Terms] OR ("sensitivity"[All Fields] AND "specificity"[All Fields]) OR "sensitivity and specificity"[All Fields] OR "specificity"[All Fields] OR "specific"[All Fields] OR "specifically"[All Fields] OR "specification"[All Fields] OR "specifications"[All Fields] OR "specificities"[All Fields] OR "specifics"[All Fields] OR "specifities"[All Fields] OR "specifity"[All Fields]) AND ("death"[MeSH Terms] OR "death"[All Fields] OR "deaths"[All Fields])) OR ("mortality"[MeSH Terms] OR "mortality"[All Fields] OR ("rate"[All Fields] AND "case"[All Fields] AND "fatality"[All Fields]) OR "rate case fatality"[All Fields]) OR ("mortality"[MeSH Terms] OR "mortality"[All Fields] OR ("rate"[All Fields] AND "crude"[All Fields] AND "death"[All Fields]) OR "rate crude death"[All Fields]) OR ("mortality"[MeSH Terms] OR "mortality"[All Fields] OR ("rate"[All Fields] AND "crude"[All Fields] AND "mortality"[All Fields]) OR "rate crude mortality"[All Fields]) OR ("mortality"[MeSH Terms] OR "mortality"[All Fields] OR ("rate"[All Fields] AND "death"[All Fields]) OR "rate death"[All Fields]) OR ("mortality"[MeSH Terms] OR "mortality"[All Fields] OR ("rate"[All Fields] AND "mortality"[All Fields]) OR "rate mortality"[All Fields]) OR ("Rates"[All Fields] AND "age specific"[All Fields] AND ("death"[MeSH Terms] OR "death"[All Fields] OR "deaths"[All Fields])) OR ("mortality"[MeSH Terms] OR "mortality"[All Fields] OR ("Rates"[All Fields] AND "case"[All Fields] AND "fatality"[All Fields]) OR "rates case fatality"[All Fields]) OR ("Rates"[All Fields] AND ("death"[MeSH Terms] OR "death"[All Fields] OR "deaths"[All Fields])) OR ("Rates"[All Fields] AND ("mortality"[MeSH Terms] OR "mortality"[All Fields] OR "mortalities"[All Fields] OR "mortality"[MeSH Subheading])) OR ("mortality"[MeSH Terms] OR "mortality"[All

Fields] OR "mortalities"[All Fields] OR "mortality"[MeSH Subheading]) OR ("postoperative complications"[MeSH Terms] OR ("postoperative"[All Fields] AND "complications"[All Fields]) OR "postoperative complications"[All Fields]) OR ("postoperative complications"[MeSH Terms] OR ("postoperative"[All Fields] AND "complications"[All Fields]) OR "postoperative complications"[All Fields] OR ("complication"[All Fields] AND "postoperative"[All Fields]) OR "complication postoperative"[All Fields]) OR ("postoperative complications"[MeSH Terms] OR ("postoperative"[All Fields] AND "complications"[All Fields]) OR "postoperative complications"[All Fields] OR ("complications"[All Fields] AND "postoperative"[All Fields]) OR "complications postoperative"[All Fields])) AND ("clinical trial"[Publication Type] OR "clinical trials as topic"[MeSH Terms] OR "clinical trial"[All Fields] OR ("clinical trial"[Publication Type] OR "clinical trials as topic"[MeSH Terms] OR "intervention study"[All Fields]) OR ("controlled clinical trial"[Publication Type] OR "controlled clinical trials as topic"[MeSH Terms] OR "controlled clinical trial"[All Fields]) OR ("randomized controlled trial"[Publication Type] OR "randomized controlled trials as topic"[MeSH Terms] OR "randomized controlled trial"[All Fields] OR "randomised controlled trial"[All Fields]))
