# Supplementary material for: Implante de Válvula Aórtica Transcateter (TAVI) versus Substituição Cirúrgica de Válvula em Resultados Clínicos em Pacientes com Estenose Aórtica
Source: Arq Bras Cardiol. 2025 Oct 28;122(10):e20240295. [Article in Portuguese] doi: 10.36660/abc.20240295 (PMC12677903; doi:10.36660/abc.20240295)
Supplement: Translation

# Translation: Transcatheter Aortic Valve Implantation (TAVI) Versus Surgical Valve Replacement on Clinical Outcomes in Patients with Aortic Stenosis

**DOI:** 10.36660/abc.20240295i

**Authors:** André Luiz Lisboa Cordeiro, Emmanuel de Souza Gonçalves, Rute Macêdo de Santana, Tayane Siqueira Martins dos Santos

**Affiliations:** 1 Brazil Centro Universitário de Excelência, Feira de Santana, BA – Brazil; 2 Brazil Escola Bahiana de Medicina e Saúde Pública, Salvador, BA – Brazil

**Keywords:** Aortic Valve Stenosis, Transcatheter Aortic Valve Replacement

## Abstract

Aortic stenosis (AS) is one of the most prevalent valve lesions, and transcatheter aortic valve replacement (TAVI) has emerged as an alternative to surgical aortic valve replacement (SAVR). TAVI is a minimally invasive procedure that has proven to be a safer option in several aspects. The objective is to review the impact of TAVI compared to surgical aortic valve replacement on mortality, postoperative complications, hospitalization, and quality of life in patients with AS. A systematic review was conducted using the PICO strategy, with searches in the PubMed, Central, and LILACS databases, employing the following descriptors: aortic stenosis, hospitalization, mortality, clinical trial, transcatheter aortic valve implantation, quality of life, postoperative complications, combined with the Boolean operators “AND” and “OR.” A total of 29 articles were found after reading the titles and abstracts. Of these, nine showed lower mortality in patients undergoing TAVI, while three reported lower mortality in the SAVR group. Eight articles had similar findings regarding complications, with six showing a lower incidence of postoperative complications in TAVI and three in SAVR. Additionally, three articles showed better quality of life in TAVI patients, while one study indicated a shorter hospital stay for TAVI patients. In all studies, the patients analyzed were over 70 years old. TAVI reduced mortality compared to SAVR in patients with AS. Furthermore, TAVI was associated with a reduction in hospital stay and improvement in quality of life. Regarding postoperative complications, the results indicated that TAVI tends to have a lower complication rate, although variations exist between studies.

## Introduction

The transcatheter aortic valve implantation (TAVI) procedure was first performed in 2002, representing a major advancement in the treatment of aortic stenosis (AS). Since its inception, the technique has transformed the management of AS, initially developed for high-risk surgical patients but gradually expanding to intermediate and even low-risk patients, providing a minimally invasive alternative to conventional surgical aortic valve replacement (SAVR). Studies have shown that TAVI is effective in both high- and low-surgical risk patients, resulting in significant improvements in clinical outcomes, including reduced mortality and morbidity associated with severe AS.^1 , 2^TAVI involves the implantation of a stent, which can be self-expanding, into the aortic annulus, covered with three internal leaflets (typically made from bovine or porcine pericardium), which function as semilunar valves. Access for the procedure is typically via catheterization of the femoral or subclavian arteries.

Over the past 20 years, there has been continuous development of new technologies and prostheses for the TAVI procedure, leading to greater reliability in valve implantation and improved long-term outcomes.^3^Operator experience has also played a critical role in the success of TAVI, and, combined with advancements in imaging techniques and procedural planning, this has facilitated the growing adoption of TAVI for the treatment of aortic stenosis. Currently, TAVI accounts for approximately 12.5% of all aortic valve replacements performed worldwide.^4^Moreover, studies have shown that, in selected patients, TAVI yields superior outcomes compared to SAVR, particularly when performed via transfemoral access.^3 , 5^

Despite the proven benefits of TAVI, uncertainties persist regarding some key outcomes of the procedure. For instance, the occurrence of stroke remains a significant concern, as valve positioning and implantation may increase the risk of embolic events.^6^Additionally, the comparison of mortality between TAVI and SAVR remains controversial, with questions surrounding the validity of tools like the Society of Thoracic Surgeons Risk Score (STS), which has not yet been fully established as a reliable indicator in all cases.^7^These issues underscore the need for a critical and thorough evaluation of the available clinical data to understand better the outcomes associated with TAVI and its true applicability across different patient populations.

Thus, this systematic review aims to analyze and compare the available data on clinical outcomes between TAVI and SAVR, with the goal of clarifying aspects that remain undefined, such as mortality, postoperative complications, hospitalization duration, and quality of life. In doing so, this review seeks to shed light on the true impact of TAVI on clinical outcomes and provide a more robust evidence base to support decision-making in clinical practice.

## Methods

### Protocol and registration

This systematic review was completed in accordance with the Preferred Reporting Items for Systematic Reviews and Meta-analyses (PRISMA) guidelines.^8^It is registered with PROSPERO (International Prospective Register of Systematic Reviews) under number CRD42021218265.

### Eligibility criteria

This systematic review followed the PICOS^9^strategy to structure the analysis of relevant studies. The study population comprised patients diagnosed with aortic dysfunction, specifically those with aortic stenosis or regurgitation, who were candidates for intervention. The primary intervention assessed was Transcatheter Aortic Valve Implantation (TAVI), a less invasive procedure compared to traditional surgical options. TAVI was directly compared with conventional treatments, which typically involve median sternotomy or thoracotomy for aortic valve replacement, both of which are more invasive surgical approaches.

The review focused on several key outcomes: the length of hospital stay, the duration of intensive care unit (ICU) stay, quality of life after the procedure, mortality rates, and the occurrence of postoperative complications, including but not limited to stroke, bleeding, and valve-related issues. These outcomes were selected due to their clinical relevance in determining the overall efficacy and safety of TAVI compared to traditional surgical interventions.

Only randomized clinical trials (RCTs) were included to ensure the highest level of evidence for the comparisons. There were no restrictions on the year or language of publication, allowing for a comprehensive inclusion of studies across different periods and regions. However, to improve the precision of the review, further elaboration on the specific inclusion and exclusion criteria for studies would strengthen the transparency of the selection process. This would help readers understand the scope of the evidence, the generalizability of the results, and any potential limitations related to the selected studies, such as sample size or methodological quality. Additionally, clarifying how the studies were assessed for bias and risk of methodological flaws would offer valuable insights into the reliability of the findings presented in the review.

### Sources of information

We conducted a computer search, consulting PubMed, Central Register of Systematic Reviews (CENTRAL), and LILACS. We also searched the list of references of previous systematic reviews and clinical trials eligible for this review. The search for articles ended in March 2024.

### Search

The search was based on the previously described PICOS^9^strategy and the Boolean operators AND and OR. We used as descriptors for the population (Aortic Stenosis or Aortic Valve Stenosis or Stenosis, Aortic or Stenosis, Aortic Valve or Stenosis, Aortic or Stenosis, Aortic Valve or Valve Stenosis, Aortic or Valve Stenosis, Aortic or Aortic Valve Stenosis) with aortic dysfunction. For the intervention (Transcatheter Aortic Valve Implantation or Transcatheter Aortic Valve Replacement) with TAVI. For comparison (conventional treatment or sternotomy, or thoracotomy). For the results (Hospitalizations or Hospital Stay or Hospital Stays or Stay Length or Stay Lengths or Stay, Hospital or Stays, Hospital or Length of Stay or Intensive Care Units or Intensive or Care Unit, Intensive or Care Units, Intensive or ICU Intensive Care Units or Unit, Intensive Care or Units, Intensive Care or Quality of Life or Health-Related Quality of Life or Health-Related Quality of Life or HRQOL or Quality of Life or Age-Specific Mortality Rate or Case Fatality Rate or Case Fatality Rate or Case Fatality Rate CFR or Crude Mortality Rate or Age-Specific Mortality Rate or Crude Mortality Rate or Mortality Rate or Mortality rates, age-specific or decline, mortality or decline, mortality or determinant, mortality or determinant, mortality or mortality differential or mortality excess or mortality excess or mortality or mortality, mortality differential or mortality, mortality excess or decline or mortality decline or mortality determinant or mortality determinant or mortality rate or mortality rate, crude or mortality or mortality rates, Differential or Mortality, Excess or Rate, Age- Specific Death or Rate, Case Fatality or Rate, Crude Death or Rate, Crude Mortality or Rate, Death or Rate, Mortality or Rates, Age-Specific Death or Rates, Case Fatality or Rates, Death or Rates, Mortality or Mortality or Postoperative Complications or Complication, Postoperative or Complications, Postoperative)were by length of hospital stay and intensive care unit; quality of life; mortality and postoperative complications. As descriptors for the study design (Clinical Trial or Intervention Study or Controlled Clinical Trial, or Randomized Controlled Trial), we used randomized clinical trials, clinical trials, and controlled trials. The search strategy developed is presented in the form of a supplement.

### Study Selection

This systematic review included randomized clinical trials involving patients who underwent TAVI. To be eligible, the clinical trial had to involve patients with aortic dysfunction undergoing TAVI. Studies with adults (18 years or older), regardless of gender, were included. The exclusion criteria were studies involving patients in palliative care, with a life expectancy of less than one year, or with a low probability of improvement in quality of life with treatment.

### Data collection process

The extraction of selected articles was performed in three sequential stages:

**1. First Stage:** Checking the titles of the studies to identify relevance.

**2. Second Stage:** Analyzing the abstracts of the articles for a more detailed pre-selection.

**3. Third Stage:** Full reading of the articles for in-depth evaluation.

After the selection, an exploratory reading of the studies was conducted, followed by a selective and analytical reading. The data extracted from the articles was organized and summarized in terms of: authors, journal, year of publication, title, and conclusions. This allowed the collection of the most relevant information for the research.

### Data items

Three authors independently (EG, RM and TS) extracted the data from the published reports using standard data extraction considering: (1) aspects of the study population, such as mean age, gender, number of patients, diagnosis; (2) aspects of the intervention performed; (3) follow-up; (4) loss to follow-up; (5) outcome measures; and (6) results presented.

### Bias and quality assessment

The risk of bias was evaluated using the Cochrane Collaboration Risk of Bias 2 tool. The domains assessed were selective outcome reporting, incomplete outcome data, blinding of outcome assessment, blinding of participants and personnel, sequence generation: allocation concealment, and other issues. The risk of bias was categorised into low, high, or uncertain risk.

## Results

According to the data presented in the article selection flowchart, the initial search of the databases yielded a total of 547 articles. Of these, 475 were excluded after reading the titles. Subsequently, 72 articles were evaluated based on their abstracts, and 14 were deemed not directly related to the subject of this study. Thus, 58 articles were selected for full-text review. Of these, 5 were excluded due to duplication, 18 were not clinical trials, 2 did not follow the required procedure, and 4 did not meet the inclusion criteria for the methods. Therefore, this systematic review includes 29 articles that met the eligibility criteria for inclusion ( Figure 1 ).

**Figure 1:** – Flowchart of the research strategy.

### Risk of bias

Risk of bias was assessed for 8 trials ( Table 1 ). Each trial was graded as either “high risk”, “low risk”, or “some concerns” ( Figure 2 ).

**Table 1:** – Baseline characteristics of selected studies

Study (Author/year)	Country	Sample	Design	Inclusion criteria	Interventions			Measurements	Results
					Intervention	Control			
	USA	742 TAVI (n = 389) SAVR (n = 353)	Randomized clinical trial	Patients with severe aortic stenosis and heart failure symptoms of the New York Heart Association (NYHA) class II	TAVI	SAVR conventional open-heart techniques with the use of cardiopulmonary bypass.	Mortality		Mortality At 1 year: 14.2% in the TAVI group 19.1% in the SAVR group Absolute reduction in risk of 4.9 percentage points (confidence interval, −0.4; P<0.001 for noninferiority; P = 0.04 for superiority).
Adams et al. 2014^10^									
Søndergaard et al. 2016^11^	Denmark and Sweden	276 TAVI (n = 142) SAVR (n = 134)	Randomized clinical trial	Severe native aortic valve stenosis	TF-TAVI	SAVR	Mortality		Mortality At 2 years 8.0% in the TAVI group 9.8% in the SAVR group p=0.54.
Skelding et al. 2016^12^	USA	353 TAVI (n = 183) SAVR (n = 170)	Randomized clinical trial	Female patients with New York Heart Association (NYHA) class II or greater symptoms and severe aortic stenosis	TAVI	SAVR conventional surgery	Mortality and Postoperative Complications		Mortality at 30 days All-cause mortality 3.8% in the TAVI group 4.7% in the SAVR group p = 0.70 Stroke: 5.6% in the TAVI group 6.5% in the SAVR group p = 0.73 Myocardial infarction: 1.6% in the TAVI group 0.6% in the SAVR group p = 0.35 Major vascular complications 7.7% in the TAVI group 0.6% in the SAVR group p = 0.001 Cardiac perforation 2.7% in the TAVI group 0.0% in the SAVR group p = 0.03 Permanent pacemaker implantation 20.7% in the TAVI group 7.2% in the SAVR group p < 0.001
Grayburn et al. 2018^13^	USA	739 TAVI (n = 386) SAVR (n = 353)	Randomized clinical trial	Patients were stratified by the severity of baseline AR (aortic regurgitation) into those with none and/or trace and those with mild AR	TAVI	SAVR	Mortality		Mortality 49% (84 of 172 patients) in the TAVI group 8% (13 of 162 patients) in the SAVR group
Conte et al. 2017^14^	USA	790 TAVI (n = 391) SARV (n = 359)	Randomized clinical trial	Severe aortic stenosis	TAVI iliofemoral or non-iliofemoral access ports	SAVR	Postoperative Complications, mortality		Mortality at 2 years 8.0% in the TAVI group 9.8% in the SAVR group p = 0.54 Major Vascular Complications: Significantly higher in the TAVI group during the periprocedural period (5.4% vs. 1.4%, p = 0.003)
Leon et al. 2021^15^	USA	950 TAVI (n = 496) Surgery (n = 454)	Randomized clinical trial	Patients with severe aortic stenosis	TAVI transfemoral	Minimally invasive surgery	Mortality		Mortality at 2 years TAVI: 57 patients (11.5%) SAVR: 78 patients (17.4%) p = 0.007
Durko et al. 2018^16^	Europe, USA, Canada	1660 TAVI (n = 864) Surgery (n = 796)	Randomized controlled trials	Patients with severe, symptomatic aortic stenosis at intermediate surgical risk	TAVI	SAVR conventional surgery	Complications		Early strokes: TAVI: 3.3% SAVR: 5.4% p = 0.031 Early encephalopathy: TAVI: 1.6% SAVR: 7.8% p < 0.001
Thyregod et al. 2013^17^	Scandinavia	280 TAVI (n = 140) SAVR (n = 140)	Randomized clinical trial	Patients 70 years or older with severe degenerative AV stenosis with symptoms or without symptoms but with left ventricular systolic dysfunction and/or hypertrophy	TAVI	SAVR complete median sternotomy	Complications or all-cause mortality (primary outcome)		Myocardial infarction, stroke, or all-cause mortality. SAVR group: 15% incidence TAVI group: 5% incidence p<0.001
Gleason et al. 2016^18^	USA	750 TAVI (n = 391) SAVR (n = 359)	Randomized clinical trial	High-risk patients (predicted SAVR mortality 15%) with severe aortic stenosis	TAVI	SAVR	Mortality and complications		30-day stroke rate: TAVI: 4.9% SAVR: 6.2% p = 0.46 1-year stroke rate: TAVI: 8.7% SAVR: 12.5% p = 0.11 2-year stroke rate: TAVI: 10.9% SAVR: 16.6% p = 0.05 2-year all-cause mortality: TAVI: 83.3% SAVR: 54.5% p = 0.29
Kapadia et al. 2018^19^	USA	5637 TAVI (n = 4389) SAVR (n = 1248)	Randomized clinical trial	Patients with aortic stenosis	TF-TAVI	SAVR	Complications and quality of life		30-day Stroke: TF-TAVI: 5.1% SAVR: 3.7% p = 0.09 30-day Major Stroke: TF-TAVI: 3.9% SAVR: 2.2% p = 0.018 SAVR (KCCQ score): Without major stroke: 79 [53, 94] With major stroke: 64 [30, 94] p = 0.03 TF-TAVI (KCCQ score): Without major stroke: 78 [49, 96] With major stroke: 60 [8, 99] p = 0.04
Mack et al. 2015^20^	USA	699 TAVI (n = 348) SAVR (n = 351)	Randomized controlled trial	Patients with severe aortic stenosis	TF-TAVI	SAVR type of surgery not specified	Mortality		Mortality At 5 years 67.8% in the TAVI group 62.4% in the SAVR group p=0.76.
Deeb et al. 2016^21^	USA	750 TAVI (n = 391) SAVR (n = 359)	Randomized clinical trial	Patients with severe aortic stenosis	TAVI	SAVR procedure at the discretion of the cardiac surgeon	Mortality and complications		Three-year outcomes All-cause mortality or stroke 37.3% in the TAVI group 46.7% in the SAVR group p=0.006 All-cause mortality 32.9% in the TAVI group 39.1% in the SAVR group p=0.068 All stroke 12.6% in the TAVI group 19.0% in the SAVR group p=0.034 Major adverse cardiovascular or cerebrovascular events 40.2% in the TAVI group 47.9% in the SAVR group p=0.025
Kapadia et al. 2015^22^	USA	358 TAVI (n = 179) SAVR (n = 179)	Randomized clinical trial	Patients with severe symptomatic inoperable aortic stenosis (mean age 83 years, Society of Thoracic Surgeons Predicted Risk of Mortality 11·7%, 54% female).	TF-TAVI	Balloon aortic valvuloplasty	Mortality		Five-year outcomes All-cause mortality 71.8% in the TAVI group 93.6% in the SAVR group p<0.0001
Thyregod et al. 2015^23^	Denmark and Sweden	274 TAVI (n = 139) SAVR (n = 135)	Randomized clinical trial	Patients ≥70 years of age with severe degenerative aortic valve stenosis	TF-TAVI	SAVR underwent conventional open heart surgery with the use of cardiopulmonary bypass	Mortality and complications		One-year outcomes Death from any cause, stroke, or MI (primary outcome) 13.1% in the TAVI group 16.3% in the SAVR group −3.2% absolute difference; p=0.43 for superiority
Gleason et al. 2018^24^	USA	750 TAVI (n = 391) SAVR (n = 359)	Randomized clinical trial	Patients with severe, symptomatic (New York Heart Association [NYHA] functional class II or greater) aortic stenosis	TAVI	SAVR	Mortality and complications		Five-year outcomes All-cause mortality 55.3% in the TAVI group 55.4% in the SAVR group Major stroke 12.3% in the TAVI group 13.2% in the SAVR group
Toff et al. 2022^25^	UK	866 TAVI (n = 450) SAVR (n = 416)	Randomized clinical trial	Patients were aged 70 years or older with severe, symptomatic aortic stenosis and increased operative risk due to comorbidity or age.	TAVI	SAVR (midline sternotomy and minimally invasive surgery	Mortality		One-year outcomes All-cause mortality 4.6% in the TAVI group 6.6% in the surgery group Adjusted absolute risk difference −2.0% (1-sided 97.5% CI, −∞ to 1.2%)
Généreux et al. 2014^26^	USA	657 SARV (n = 313) TAVI-TF (n = 240) TARV-TA (n = 104)	Randomized clinical trial	Patients with severe symptomatic aortic stenosis	TF-TAVI OR TA-TAVI	SAVR	Postoperative Complication		30-day outcomes Major BC (blood complications) 22.7% after SAVR 11.3% after TF-TAVI 8.8% after TA-TAVI p<0.0001 30-day transfusion rate 17.9% after SAVR 7.1% after TF-TAVI 4.8% after TA-TAVI p<0.0001
Amrane et al. 2019^27^	Canada, Europe, USA	936 SARV (n = 438) TAVI (n = 498)	Randomized clinical trial	Patients with severe symptomatic AS are considered to be of intermediate surgical risk.	TF-TAVI	SAVR	Mortality		One-year outcomes All-cause mortality 6.5% after TAVI 6.7% after SAVR
Leon et al. 2016^28^	USA	2032 TAVI (n = 1011) SAVR (n = 1021)	Randomized clinical trial	Intermediate-risk patients with severe aortic stenosis	TF-TAVI OR TA-TAVI	SAVR transthoracic or transaortic access	Postoperative Complications and Mortality		Death from any cause or a disabling stroke Overall: Similar rates between TAVI and surgery p=0.001 for noninferiority
Reardon et al. 2011^29^	USA, Canada, Europe	1657 TAVI (n = 863) SAVR (n = 794)	Randomized clinical trial	Intermediate-risk patients with severe symptomatic aortic stenosis	TF-TAVI	SAVR	Postoperative Complications, life quality, and mortality		24-month outcomes All-cause mortality 11.4% in the TAVI group 11.6% in the surgery group 95% confidence interval for difference, −3.8% to 3.3% Disabling stroke Similar rates in both groups Quality of life (KCCQ summary score) Significant improvement in both groups over 24 months A greater proportion of patients improved at 1 month in the TAVI group compared to the surgery group
Smith et al. 2011^30^	EUA, Canada, Germany	699 SARV (n = 351) TAVI-TF (n = 244) TARV-TA (n = 104)	Randomized clinical trial	Patients with severe aortic stenosis and cardiac symptoms (New York Heart Association [NYHA] class II function or worse)	TF-TAVI OR TA-TAVI	SAVR conventional surgery	Postoperative Complications and mortality		30-day and 1-year outcomes All-cause death 30 days: 3.4% in transcatheter group vs. 6.5% in surgical group (p=0.07) 1 year: 24.2% in transcatheter group vs. 26.8% in surgical group (p=0.44) Reduction of 2.6 percentage points in the transcatheter group Upper limit of 95% CI for difference: 3.0 percentage points Predefined margin: 7.5 percentage points; p=0.001 for noninferiority Major stroke 30 days: 3.8% in transcatheter group vs. 2.1% in surgical group (p=0.20) 1 year: 5.1% in transcatheter group vs. 2.4% in surgical group (p=0.07) Major vascular complications 30 days: 11.0% in transcatheter group vs. 3.2% in surgical group (p<0.001)
Makkar et al. 2020^31^	USA	2032 TARV- TF (n = 1550) SAVR (n = 482)	Randomized clinical trial	Intermediate-risk patients with severe symptomatic aortic stenosis	TF-TAVI	SAVR in the transthoracic region	Mortality, Postoperative Complications, Quality Of Life		Five-year outcomes All-cause mortality 4 7.9% in the TAVI group 43.4% in the surgery group p=0.21 At least mild paravalvular aortic regurgitation 33.3% in the TAVI group 6.3% in the surgery group Health status improvement Similar improvement in both TAVI and surgery groups at 5 years
Kapadia et al. 2014^32^	USA	449 TAVI (n = 220) SAVR (n = 229)	Randomized clinical trial	Inoperable patients with severe aortic stenosis	TAVI TF	SAVR TF	Mortality		Three-year mortality 54.1% in the TAVI group 80.9% in the SAVR group p<0.001
Baron et al. 2019^33^	USA	943 TAVI (n = 494) SAVR (n = 449)	Randomized clinical trial	Patients with severe aortic stenosis (AS) of low surgical risk	Transfemoral TAVI using a balloon-expandable valve	SAVR	Quality of life		At 1 month TAVI showed better health status than SAVR Mean difference in KCCQ-OS: 16.0 points, p<0.001 At 6 months TAVI remained better than SAVR, though with a reduced effect Mean difference in KCCQ-OS: 2.6 points, p<0.04 At 12 months TAVI still showed better health status, with a further reduced effect Mean difference in KCCQ-OS: 1.8 points, p<0.04 Proportion of patients with KCCQ-OS ≥75 and no significant decline from baseline At 6 months: 90.3% in TAVI vs. 85.3% in SAVR, p=0.03 At 12 months: 87.3% in TAVI vs. 82.8% in SAVR, p=0.07
Garcia et al. 2021^34^	USA	1524 TAVI (n = 1045) SAVR (n = 479)	Randomized clinical trial	Patients with severe aortic stenosis (AS) and chronic kidney disease (CKD) undergoing TAVI or SAVR	TAVI	SAVR	Complication		Perioperative Acute Kidney Injury (AKI) 26.3% after SAVR 10.3% after TAVI p<0.001
Vandermolen et al. 2021^35^	USA	322 TAVI (n = 220) SAVR (n = 102)	Randomized clinical trial	Patients with classic low-flow, low-gradient aortic stenosis	TAVI	SAVR	Mortality		Median follow-up of 2.7 years (range 1.5–4.1 years) 99 patients had died: 70 (31.8%) in the TAVI group 29 (28.4%) in the SAVR group
Forrest et al. 2022^36^	USA	1414 TAVI (n = 730) SAVR (n = 684)	Randomized clinical trial	Patients with aortic stenosis in low-risk patients	TAVI	SAVR	Mortality and complications		All-cause mortality rates 3.5% in the TAVI group 4.4% in the SAVR group p=0.366 Disabling stroke rates 1.5% in the TAVI group 2.7% in the SAVR group , p=0.119
Kapadia et al. 2015^23^	USA	358 TAVI (n = 179) SAVR (n = 179)	Randomized clinical trial	Patients with severe inoperable aortic stenosis	TAVI	SAVR	Mortality		Five-year outcomes All-cause mortality 71.8% in the TAVI group 93.6% in the SAVR group p<0.0001
Forrest et al. 2023^37^	USA	1414 TAVI (n = 730) SAVR (n = 684)	Randomized clinical trial	Low-Risk Patients with Aortic Stenosis	TAVI with self-expanding supraannular valve	SAVR	Mortality and complications		Mortality rates (all-cause or disabling stroke) Year 1: −1.8% difference (TAVI vs. Surgery) Year 2: −2.0% difference Year 3: −2.9% difference Incidence of mild paravalvular regurgitation 20.3% in the TAVI group 2.5% in the surgery group Pacemaker placement 23.2% in the TAVI group 9.1% in surgery group p<0.001 Moderate or greater paravalvular regurgitation <1% in both groups No significant difference

TF-TAVI: transfemoral aortic valve transcatheter implantation; SAVR: surgical aortic valve replacement; AR: aortic regurgitation; NYHA: New York Heart Association Functional Classification; TA-TAVI: transfemoral aortic valve transcatheter implantation; LDB: life-threatening or disabling hemorrhage; AKI: acute kidney injury; AV: aortic valve; KCCQ: Kansas City Cardiomyopathy Questionnaire; KCCQ-OS: Kansas City Cardiomyopathy Questionnaire Overall Summary Score; HR: Hazard Ratio; CI: confidence interval; USA: United States of America; UK: United Kingdom; AS: aortic stenosis.

**Figure 2:** – Risk of bias stratification for all included studies using the Cochrane Risk of Bias Tool 2. Domains - D1: Bias resulting from the randomization process; D2: Bias due to deviations from the intended interventions; D3: Bias due to the absence of outcome data; D4: Bias in outcome measurement; D5: Bias in the selection of the reported outcome.

### Participants

A total of 19,567 patients received the intervention in the studies included in this review. The age ranged from 73 to 83 years, and the prevalence was male (58.5%). The inclusion criteria were patients with aortic dysfunction who underwent the TAVI or SAVR procedure. The other data is shown in Table 1 . Central Illustration provides a summary of the findings related to the selected articles.^10 - 37^

### Intervention

Of the studies included in this review, the CoreValve^9 , 17 , 20 , 22^was the most commonly used valve for TAVI, as it was employed in the majority of procedures. However, other valves were also reported, including the Edwards SAPIEN 1st generation,^21^the SAPIEN XT balloon-expandable heart valve,^15 , 20 , 27^and the 2nd generation SAPIEN XT, all of which are made from bovine or porcine pericardium,^18 , 19^as well as the Sapien 3 valve.^14^The preferred access route for TAVI was transfemoral; however, in some studies, transapical access was also used, albeit to a lesser extent.

## Discussion

This systematic review was initiated with the aim of evaluating the impact of TAVI on clinical outcomes, with an emphasis on mortality, hospitalization, postoperative complications, and quality of life. In this regard, a review of the studied articles showed that mortality rates were significantly lower^14 , 20 , 21 , 31 , 24 , 36^in patients undergoing TAVI, along with better prospects in terms of quality of life.^18^Furthermore, the study revealed that the rate of postoperative complications, such as bleeding and acute kidney injury, was lower in the TAVI group. It is important to clarify that the subanalysis of the included studies is not part of the analysis, so that the same patient may be counted more than once. Therefore, patient duplication in the count is possible. Another factor that must be taken into consideration is age. All selected studies investigated individuals over 70 years of age.

As shown, most of the articles demonstrate that TAVI performs better than SAVR in terms of mortality. Adams et al.^10^suggest that several factors may have contributed to TAVI’s superior performance, such as the less invasive approach, faster mobilization, and postoperative recovery, along with lower rates of strokes and other vascular complications. Additionally, the authors reported that a higher rate of paravalvular regurgitation, which is common in TAVI patients, did not impact overall survival in their study. There are other articles, such as the one by Mack et al.,^20^which argue that the substantially higher rate of paravalvular regurgitation in TAVI results in a lower survival rate for patients. However, this is not considered a factor that undermines TAVI as an alternative to conventional surgery.

Regarding the incidence of postoperative complications^13 , 14^the articles showed little difference between TAVI and SAVR. However, the study by Durko et al.^16^revealed a notable finding: the higher incidence of encephalopathy after SAVR compared to TAVI. This may be attributed to the use of cardiopulmonary bypass in SAVR, which leads to reduced oxygenation of the brain, increasing the risk of encephalopathy. Additionally, there is a lower rate of early stroke in TAVI compared to SAVR, which is related to the lower surgical risk, the technological advancements in the procedure, and the implantation technique used.

Furthermore, the study by Généreux et al.^26^showed that bleeding complications and transfusions were 2 to 3 times more frequent in the SAVR group compared to TAVI, which the author finds unsurprising, given the more invasive nature of the surgery and the well-documented coagulopathy that occurs after cardiopulmonary bypass, which supports his claim.

The study by Reardon et al.,^29^for example, showed that SAVR was associated with higher rates of acute kidney injury, atrial fibrillation, and the need for transfusion. The invasive nature of the procedure can partially explain these complications, as major surgeries tend to prolong hospital stays, mechanical ventilation times, and the need for inotropic drugs and mechanical circulatory support. However, it is important to acknowledge that patient-specific factors and surgical techniques may also influence these outcomes, and additional studies have reported varying results regarding the incidence of such complications. On the other hand, TAVI demonstrated higher rates of residual aortic regurgitation and the need for pacemaker implantation. These issues may stem from trauma, ischemia, hemorrhage, and edema at the level of the atrioventricular node, as well as from the nature of the valve itself, considering the differences between balloon-expandable and self-expanding valves. Notably, the incidence of pacemaker implantation and paravalvular regurgitation has evolved over time with technological advancements and improved implantation techniques. These findings suggest a better quality of life associated with TAVI in the first 30 days compared to SAVR. However, after six months, the quality of life appears to be comparable between the two procedures, reinforcing the need for long-term follow-up to assess durability and clinical outcomes.

Corroborating the idea mentioned above, the study by Baron et al.^33^also highlights that TAVI demonstrates superior results in terms of quality of life compared to SAVR, a finding consistent with that of Reardon et al.^29^Baron et al.^33^attribute this to the higher rates of bleeding, acute kidney injury, and postoperative atrial fibrillation in SAVR, as well as the relatively lower rates of vascular injury and paravalvular leak in TAVI. These factors are considered key to the better quality of life observed in patients undergoing TAVI. This explanation helps to justify why the KCCQ score is better in TAVI patients up to 6 months, even though this difference decreases substantially over time.

The comparison between transcatheter aortic valve replacement and conventional surgical aortic valve replacement has been a central topic in several randomized studies, especially in patients with severe aortic stenosis. The data presented by Adams et al.^10^show a significant advantage of TAVI in terms of mortality, with an absolute reduction of 4.9 percentage points in the mortality rate compared to SAVR after 1 year, with p = 0.04 for superiority. This aligns with the findings of other studies, such as Thyregod et al.,^17^which also demonstrated the superiority of TAVI in terms of severe complications and mortality. These results suggest that for certain patient groups, such as those with high surgical risk or comorbid conditions, TAVI may be a safer and more effective alternative, especially when considering postoperative recovery and reduced hospitalization time.

Over time, TAVI has evolved significantly since its introduction over two decades ago, with improvements in valve designs, procedural techniques, and patient selection criteria. Short-term benefits, including lower mortality rates and reduced complications, have been well documented. However, long-term data suggest the need for further research to assess valve durability, late complications, and functional outcomes beyond five years.

An evolutionary consideration of TAVI prosthetics can be highly valuable, as significant improvements have been made over time, particularly in reducing paravalvular leakage. Advances in valve design, materials, and delivery techniques have led to better sealing and more precise valve positioning, contributing to improved clinical outcomes and long-term durability. These enhancements have helped to address one of the key challenges of TAVI, allowing for better hemodynamic performance and a lower incidence of complications, which ultimately benefits patients undergoing this less invasive procedure.

The study presents some limitations that may impact the interpretation of the results. The inclusion of different types and brands of valves can introduce variability, making it difficult to compare the effects of each model directly. Additionally, differences in surgical techniques and protocols among the medical teams involved in the trials may affect the consistency of the results, limiting the applicability of the findings to broader clinical practices. The heterogeneity in patient populations and clinical settings may also introduce bias, making it difficult to generalize the results to all contexts. Furthermore, the inclusion criteria used may have excluded relevant data, reducing the study’s representativeness for more diverse populations.

Despite these limitations, the study’s results provide valuable insights for clinical practice. The different approaches and types of valves analyzed offer a broader understanding of the available options, which can be beneficial in personalizing treatments and optimizing outcomes for different patient profiles. The TAVI approach, for example, has shown clear benefits, particularly with the transfemoral technique, which may be associated with better recovery and lower surgical risk. Even with the need for caution in generalizing the results, the findings make a significant contribution to the advancement of understanding in cardiac interventions, providing a solid foundation for future investigations and improvements in clinical practices.

## Conclusion

TAVI in patients with AS reduced mortality compared to SAVR. TAVI seems to reduce the length of hospital stay, bleeding, and acute renal injury, and improve quality of life. It should be taken into account, based on the selected articles, that the elderly (over 70 years old) are the population most exposed to this type of intervention, probably due to the high surgical risk of an open procedure.

## Supplement

Search strategy in PubMed

## Supplementar Material

*Supplemental Materials For additional information, please click here.
